# Effects of fertilization on microbial abundance and emissions of greenhouse gases (CH_4_ and N_2_O) in rice paddy fields

**DOI:** 10.1002/ece3.1879

**Published:** 2016-01-22

**Authors:** Xianfang Fan, Haiyang Yu, Qinyan Wu, Jing Ma, Hua Xu, Jinghui Yang, Yiqing Zhuang

**Affiliations:** ^1^ State Key Laboratory of Soil and Sustainable AgricultureInstitute of Soil ScienceChinese Academy of SciencesNanjing210008China; ^2^ University of the Chinese Academy of SciencesBeijing100039China; ^3^ Zhenjiang Institute of Agricultural ScienceJurong212400China

**Keywords:** *amoA*, CH_4_, *mcrA*, N_2_O, *nirK*, *nirS*, *nosZ*, *pmoA*

## Abstract

This study is to explore effects of nitrogen application and straw incorporation on abundance of relevant microbes and CH_4_ and N_2_O fluxes in a midseason aerated rice paddy field. Fluxes of CH_4_ and N_2_O were recorded, and abundance of relevant soil microbial functional genes was determined during rice‐growing season in a 6‐year‐long fertilization experiment field in China. Results indicate that application of urea significantly changed the functional microbial composition, while the influence of straw incorporation was not significant. Application of urea significantly decreased the gene abundances of archaeal *amoA* and *mcrA*, but it significantly increased the gene abundances of bacterial *amoA*. CH_4_ emission was significantly increased by fresh straw incorporation. Incorporation of burnt straw tended to increase CH_4_ emission, while the urea application had no obvious effect on CH_4_ emission. N_2_O emission was significantly increased by urea application, while fresh or burnt straw incorporation tended to decrease N_2_O emission. The functional microbial composition did not change significantly over time, although the abundances of *pmoA*, archaeal *amoA*,* nirS,* and *nosZ* genes changed significantly. The change of CH_4_ emission showed an inverse trend with the one of the N_2_O emissions over time. To some extent, the abundance of some functional genes in this study can explain CH_4_ and N_2_O emissions. However, the correlation between CH_4_ and N_2_O emissions and the abundance of related functional genes was not significant. Environmental factors, such as soil Eh, may be more related to CH_4_ and N_2_O emissions.

## Introduction

Both methane (CH_4_) and nitrous oxide (N_2_O) are recognized as potent greenhouse gases, 25 and 298 times higher than carbon dioxide (CO_2_), respectively, in global warming potential (IPCC, 2007). Furthermore, CH_4_ affects the chemistry and oxidation capacity of the atmosphere, and N_2_O participates in destruction of the stratospheric ozone layer (IPCC, 2007). Rice paddy soils are known to be a significant anthropogenic source of CH_4_ and N_2_O (Ma et al. 2007). Fluxes of CH_4_ and N_2_O from rice paddy fields vary depending strongly on agricultural management. Fertilizer nitrogen application and straw incorporation into the soil are two common soil fertility management practices in rice cultivation. Nitrogen application into paddy soils enhances N_2_O emission from the fields (Ma et al. 2007; Pittelkow et al. 2014), although its effect on CH_4_ emission from the fields was contradictory (Banger et al. 2012). However, straw incorporation into paddy soils increases CH_4_ emission by 110.7 ± 1.2%, but decreases N_2_O emission by 15.2 ± 1.1% (Liu et al. 2014).

In rice paddy fields, soil microbial activities are the main factors controlling production and uptake of CH_4_ and N_2_O (Conrad 1996). Methanogenic archaea that produce CH_4_ and methanotrophic bacteria that oxidize a portion of it largely control CH_4_ emission from rice paddy fields (Conrad 2007), while microbial nitrification and denitrification contribute approximately 70% of N_2_O emission from rice paddy fields (Braker and Conrad 2011). Yet, surprisingly little is known about the interactions between the agro‐ecosystem management practices and the soil microbial community (Staley and Reysenbach 2002). Moreover, to our knowledge, no reports are available in the literature about any in‐situ rigorous assessment of the soil microbial community coupled with rigorous measurement of CH_4_ and N_2_O emission from rice paddy fields. There are only a few discussing relationships between N_2_O emission and related soil microbial community in upland soils. However, the effects of related soil microbial structure and abundance on N_2_O emission are inconsistent in different studies. Some studies indicate N_2_O emission is strongly linked to bacterial functional group abundance (Morales et al. 2010; Cantarel et al. 2012), while the others suggest the N_2_O emissions is more related to changes in soil environmental conditions but not the size of the functional microbial community (Attard et al. 2011; Vermue et al. 2013).

In this study, efforts are made to investigate the relationship between field CH_4_ and N_2_O fluxes and soil microbial abundance as affected by agricultural management practice in an intermittently irrigated rice paddy field, in an attempt to answer the following questions: (1) How do the abundance of soil microbial methanogen/methanotroph and nitrifier/denitrifier responds to different agricultural management practices? (2) Are variations in field CH_4_ and N_2_O fluxes mirrored by changes in microbial community of some specific soil microbial functional groups?

## Materials and Methods

### Experimental site and sampling

The long‐term fertilization field experiment site was a tract of rice paddy field, located at Jurong (31°58′N, 119°18′E), Jiangsu Province, China. The experiment started in 2006 and has been following a common rice–wheat rotation system prevailing in the region ever since. The soil of the experiment field is classified as Typic Haplaquepts (USDA Taxonomy 1975). Soil pH (H_2_O) was 5.9, and total C and N were 0.82% and 0.11%, respectively (Ma et al., 2009).

The experiment was designed to have four treatments and three replicates for each treatment laid out in a randomized plot pattern: Treatment CK (without fertilizers), Treatment N (urea applied), Treatment NFS (urea application + fresh straw incorporation), and Treatment NBS (urea + burnt straw applied). The prilled urea (1.5 mm grains) was broadcast to the plots as practiced by most of the local farmers. In Treatment NFS, dry wheat straw (C/N = 85) was chopped into sections 1 cm in length, spread evenly over the field and then plowed into the topsoil (0–15 cm) layer. In Treatment NBS, wheat straw was broadcast evenly over the field and burned in situ. Rice seedlings (Huajing 3), 32 days old, were transplanted on 26 June into all the plots, and the crop was harvested on 30 October. Midseason aeration started on 30 July, and lasted till 8 Aug. The same fertilization and water management practices were adopted for all the treatments except CK in the experiment as in the local rice cultivation. For details, please refer to Table S1.

Soil samples were collected from each plot at each of the four rice growth stages, that is, on July 10th for the seeding stage, on July 24th for the tillering stage, on August 20th for the booting stage, and on October 6th for the ripening stage in 2012. Three soil cores were collected from each plot using a stainless steel corer, 7 cm in inner diameter and 25 cm in length, and then prepared into mixture on a plot basis. The mixture samples were packed into sterile plastic bags, separately, which were then sealed and placed on ice until transported to the laboratory. All the soil samples for analysis of soil properties were stored at 4°C and those for DNA extraction at −20°C.

Soil Eh was measured using Pt‐tipped electrodes (Hirose Rika Co. Ltd. Niwa‐Gun, Aichi, Japan) and an oxidation–reduction potential meter (PRN‐41, DKK.TOA, Tokyo, Japan). To measure soil Eh, the electrodes were inserted into the soil at a depth of 10 cm and maintained throughout rice‐growing period. All soil Eh measurements were made in triplicate. Effect of soil temperature and pH on Eh was not considered (Ma et al. 2008). Nitrate and ammonia were extracted with 2 molL^−1^ potassium chloride and determined with a Continuous Flow Analyzer (SAN++; Skalar, Holland, the Netherlands).

### Sampling and measurement of field CH_4_ and N_2_O fluxes

Fluxes of CH_4_ and N_2_O were monitored with the closed chamber method. The flux chambers (0.5 m long × 0.5 m wide × 1 m high) were made of plexiglas and equipped with a fan inside to ensure complete gas mixing. Plastic bases for the chambers were installed before rice transplantation in all the plots, and kept there until rice harvest. Removable wooden boardwalks (2 m long) were set up to avoid soil disturbances during sampling and measuring. Gas samples were generally collected once every 4 days before September 2nd, and sampling frequency was changed as once every 7 days after September 2nd. The four gas samples from each chamber were collected using 18‐mL vacuum vials at 15 min intervals between 08:30 and 11:30 am on sampling days (Zhang et al. 2012). Concentrations of CH_4_ in the air samples collected from the chambers were analyzed with a gas chromatograph (Shimadzu GC‐12A, Kyoto, Japan) equipped with a flame ionization detector, and concentrations of N_2_O were analyzed with a gas chromatograph (Shimadzu GC‐14B) equipped with an electron capture detector. CH_4_ and N_2_O fluxes were determined from the slope of linear regression and expressed in mg CH_4_ m^−2^^−^h and *μ*g N_2_O‐N m^−2^^−^h, respectively.

### DNA extraction

Before DNA extraction, freeze‐drying of the sediment samples at −53°C was performed in a freeze dryer (Lyophilizer, FreeZone 2.5 L Benchtop Freeze Dry System; Labconco, Kansas City, MO). DNA was extracted from the five layers sediment in three replicates, using a FastDNA spin kit for soil (MP Biomedicals LLC; Solon, OH, USA). And according to the instructions, 0.5 g dried sediment was used for the DNA extraction. Soil DNA was further purified and dissolved with 50 *μ*L elution buffer. Quality and size of soil DNAs were checked by electrophoresis on 1% agarose. Soil DNA quantity and purity were determined with a NanoDrop ND‐1000 UV–Vis Spectrophotometer (NanoDrop Technologies, Wilmington, DE).

### Real‐time qPCR assays

Abundance of the related soil microbes was estimated by quantitative PCR (qPCR) of genes encoding the catalytic subunit of the enzymes key to CH_4_ generation and oxidation pathways, nitrification and denitrification pathways.

Fragments of the genes encoding the methyl coenzyme‐M reductase (*mcrA*), the *α* subunit of the particulate methane monooxygenase (*pmoA*), the ammonia monooxygenase (*amoA*), the cytochrome *cd*_1_ nitrite reductases (*nirS*), the copper nitrite reductases (*nirK*), and the N_2_O reductases (*nosZ*) were amplified using primers and thermal cycling conditions described previously. For detailed sequences and limitations of the primer sets, please refer to Table S2 and Table S3, respectively.

Real‐time quantitative PCR was performed using a CFX96 Optical Real‐Time Detection System (Bio‐Rad Laboratories, Inc. Hercules, CA). Quantification was based on the fluorescence intensity of the SYBR Green dye, which binds to double‐stranded DNA. Standard curves were obtained by means of serial dilutions of linearized plasmids containing cloned genes of *mcrA*,* pmoA*,* amoA*,* nirS*,* nirK,* and *nosZ* amplified from archaeal or bacterial strains. Blank was always run with water as template instead of soil DNA extracted. The real‐time PCR assay was carried out in 10‐mL reaction volume containing 5 *μ*L of SYBR^®^ Premix Ex Taq (TaKaRa Biotech, Dalian, China), 0.25 *μ*molL^−1^ of each primer, and 1.0 *μ*L template. Specific amplification was verified by melting curve analysis, which always results in a single peak.

### Statistical analysis

The normality and homogeneity of data were tested before analysis. Data were *Z*‐standardized (mean = 0, standard deviation = 1).

One‐way ANOVA with Duncan's post hoc tests was performed to evaluate the differences between the treatments on each sampling day at the significant level of 0.05. Pearson correlation was carried out between CH_4_ and N_2_O emissions and the soil microbial gene abundances, physicochemical factors using the data of all the four time points across all treatments. Effects of management practices and time on abundance of the related soil microbial genes and physicochemical factors were analyzed with the one‐way ANOVA analysis method using the data of all the 4 time points across all treatments. The analyses above were performed using SPSS version 16.0 (SPSS Inc., Chicago, IL).

An analysis of similarities (ANOSIM) on Bray–Curtis dissimilarity matrices was conducted to test whether there was indeed a significant difference in functional microbial composition among different treatments. Principal component analysis (PCA) was performed on the relative abundance of the functional genes to visually interpret community dissimilarity. To investigate the relationship between the functional microbial composition and physicochemical factors, multivariate constrained ordination method was used. Detrended correspondence analysis (DCA) showed a short environmental gradient (largest axis length = 0.18). Consequently, redundancy analysis (RDA) was performed. The significance of physicochemical factors was tested with Monte Carlo permutations. The analyses above were conducted in R for statistical computing (R Development Core Team, 2013), using the vegan package (Oksanen et al. 2013). For more details of RDA, please refer to Table S4.

## Results

### Abundance of related soil microbial functional genes

**Figure 1:**
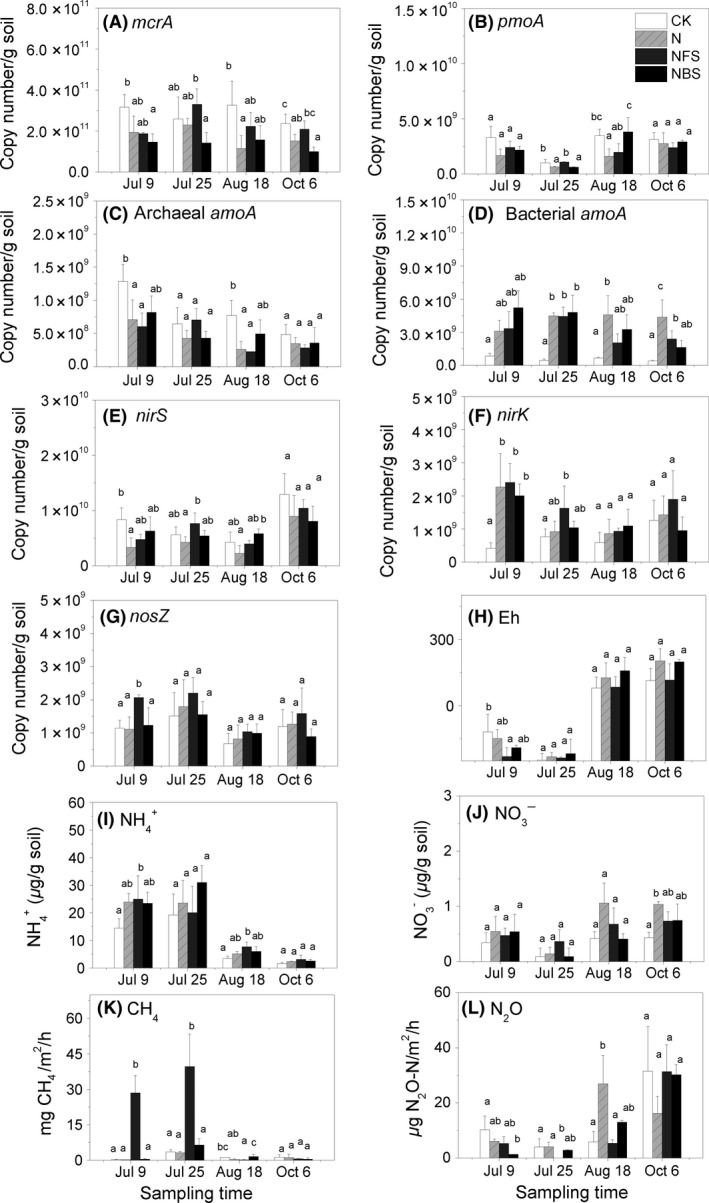
Abundance of functional genes, biochemical parameters, and fluxes of CH_4_ & N_2_O at the four sampling times (July 10th, July 24th, August 20th, October 6th): (A) *mcrA*, (B) *pmoA*, (C) archaeal *amoA*, (D) bacterial *amoA*, (E) *nirS*, (F) *nirK* (G) *nosZ*, (H) Eh, (I) ${\text{NH}}_{4}^{+}$, (J) ${\text{NO}}_{3}^{-}$, (K) CH_4_, (L) N_2_O. CK: without fertilizers, N: urea, NFS: urea + fresh straw, NBS: urea + burnt straw.

The abundance of *mcrA* gene did not change significantly over time. The abundance of *mcrA* gene was significantly lower in Treatment N and Treatment NBS, compared with Treatment CK. However, the abundance of *mcrA* gene was not significantly different between Treatment CK and Treatment NFS (Fig. 1A, Table 2).

The abundance of *pmoA* gene changed significantly over time. The abundance of *pmoA* gene tended to be lower in Treatment N, compared with Treatment CK. However, the abundance of *pmoA* gene was not obviously different among Treatment CK, Treatment NFS, and Treatment NBS (Fig. 1B, Table 2).

The abundance of archaeal *amoA* gene changed significantly over time. The abundance of bacterial *amoA* did not change significantly over time. Urea application significantly decreased the gene abundance of archaeal *amoA*, regardless of addition of straw or not (Fig. 1C, Table 2). Contrary to that, urea application significantly increased the gene abundance of bacterial *amoA*, regardless of addition of straw or not (Fig. 1D, Table 2).

The abundance of *nirS* gene changed significantly over time. The abundance of bacterial *nirK* did not change significantly over time. Urea application without straw significantly decreased the gene abundance of *nirS*, while urea application with straw tended to decrease the gene abundance of *nirS* (Fig. 1E, Table 2). Contrary to that, urea application tended to increase the gene abundance of *nirK*, regardless of addition of straw or not (Fig. 1F, Table 2).

The abundance of *nosZ* gene changed significantly over time. Urea application with fresh straw significantly increased the gene abundance of *nosZ* (Fig. 1G, Table 2). However, the abundance of *nosZ* gene was not significantly different among Treatment CK, Treatment N, and Treatment NBS.

### Soil physiochemical parameters

Soil Eh was significantly higher after midseason aeration, changing significantly over time. Soil Eh tended to be lower in Treatment NFS, compared with Treatment N and Treatment NBS. Soil Eh tended to be higher in Treatment N and Treatment NBS, compared with Treatment CK (Fig. 1H, Table 2).

Significantly lower soil ${\text{NO}}_{3}^{-}$ contents were found before midseason aeration, while significantly lower soil ${\text{NH}}_{4}^{+}$ contents were found after midseason aeration, both changing significantly over time. Urea application tended to increase soil ${\text{NH}}_{4}^{+}$ and ${\text{NO}}_{3}^{-}$ contents, regardless of addition of straw or not (Fig. 1I–J).

### CH_4_ and N_2_O fluxes

**Figure 2:**
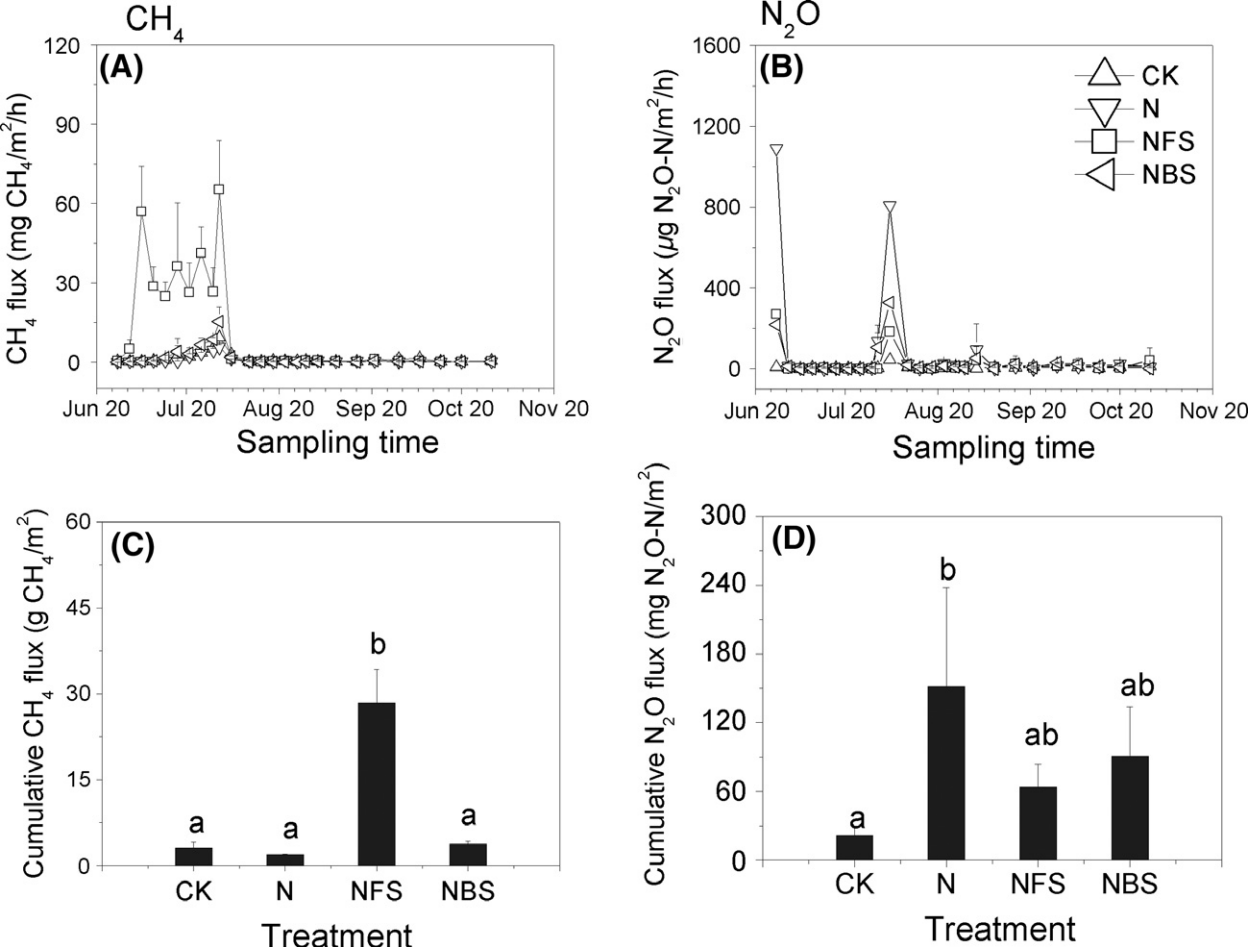
Flux variation of (A) CH_4_ and (B) N_2_O. Cumulated flux of rice season: (C) CH_4_ and (D) N_2_O. CK: without fertilizers, N: urea, NFS: urea + fresh straw, NBS: urea + burnt straw.

Emission of CH_4_ and N_2_O varied significantly over time. Emission of CH_4_ was significantly higher before midseason aeration, while emission of N_2_O was significantly lower before midseason aeration (Figs. 1K–L, 2A and B). Urea application with fresh straw significantly increased cumulative CH_4_ emission. However, compared with Treatment CK, urea application had no obvious effect on cumulative CH_4_ emission, while urea application with burnt straw tended to increased it (Fig. 2C).

Contrary to cumulative CH_4_ emission, cumulative N_2_O emission was significantly increased by urea application without straw. Additionally, urea application with straw tended to increased cumulative N_2_O emission. However, compared with Treatment N, the cumulative N_2_O emission tended to be lower in Treatment NFS and Treatment NBS (Fig. 2D).

### Statistical analysis

The result of correlation analysis showed soil Eh was significantly related to CH_4_ and N_2_O emission. However, the correlation between the functional gene abundance and CH_4_ and N_2_O emission was not significant (Table 1). ANOVA analysis indicates that urea application without straw significantly affected the abundance of *mcrA*, archaeal *amoA*, bacterial *amoA,* and *nirS* genes. Urea application with fresh straw significantly affected the abundances of archaeal *amoA*, bacterial *amoA,* and *nosZ* genes. Urea application with burnt straw significantly affected the abundances of *mcrA*, archaeal *amoA,* and bacterial *amoA* genes. Time significantly affected the abundances of *pmoA*, archaeal *amoA*,* nirS,* and *nosZ* genes and all the three environmental factors (Table 2). The result of ANOSIM showed urea application significantly affected the functional microbial composition (significance level = 0.1%), regardless of straw incorporation or not, compared with the control one. Besides, the functional microbial composition in Treatment NFS was significantly different from the one in Treatment NBS (significance level = 1.4%), while no significant differences were observed between Treatment N and Treatment NFS or between Treatment N and Treatment NBS (Table 3).

**Table 1:** Correlation between CH_4_ and N_2_O emission and the soil physiochemical parameters and the abundance of functional genes (Pearson two‐tailed test, *n* = 30)

Indicator	CH_4_	N_2_O
*mcrA*	0.031	−0.247
*pmoA*	−0.191	0.421*
*amoA*‐AOA	0.022	−0.295
*amoA*‐AOB	0.158	−0.298
*nirS*	−0.110	0.373*
*nirK*	0.431*	−0.076
*nosZ*	0.471**	−0.325
Eh	−0.462*	0.727**
${\text{NH}}_{4}^{+}$	0.507**	−0.682**
${\text{NO}}_{3}^{-}$	−0.198	0.432*

**Indicates the correlation is significant at the 0.01 level; *Indicates the correlation is significant at the 0.05 level.

**Table 2:** The effects of management practices on the related microbial gene abundances and the physicochemical parameters by ANOVA analysis (significant values are shown in bold)

Variable	Factor			
	N	N + Fresh straw	N + Burnt straw	Time
*mcrA*	**0.001**	0.155	**0.002**	0.545
*pmoA*	0.081	0.441	0.279	**<0.001**
Archaeal *amoA*	**0.001**	**0.005**	**0.041**	**0.004**
Bacterial *amoA*	**<0.001**	**0.001**	**<0.001**	0.153
*nirS*	**0.041**	0.421	0.188	**<0.001**
*nirK*	0.208	0.092	0.102	0.276
*nosZ*	0.813	**0.037**	0.684	**0.011**
Eh	0.443	0.964	0.770	**<0.001**
${\text{NH}}_{4}^{+}$	0.547	0.451	0.770	**<0.001**
${\text{NO}}_{3}^{-}$	0.465	0.074	0.745	**<0.001**

**Table 3:** The effects of management practices on the microbial community analyzed by ANOSIM (Number of permutations = 999, *n* = 42)

	Global *R*	Number of permuted statistics greater than or equal to global *R*	Significance level
CK‐N	0.672	0	0.1%
CK‐NFS	0.508	0	0.1%
CK‐NBS	0.773	0	0.1%
N‐NFS	0.067	158	15.9%
N‐NBS	0.047	206	20.7%
NFS‐NBS	0.212	13	1.4%

**Figure 3:**
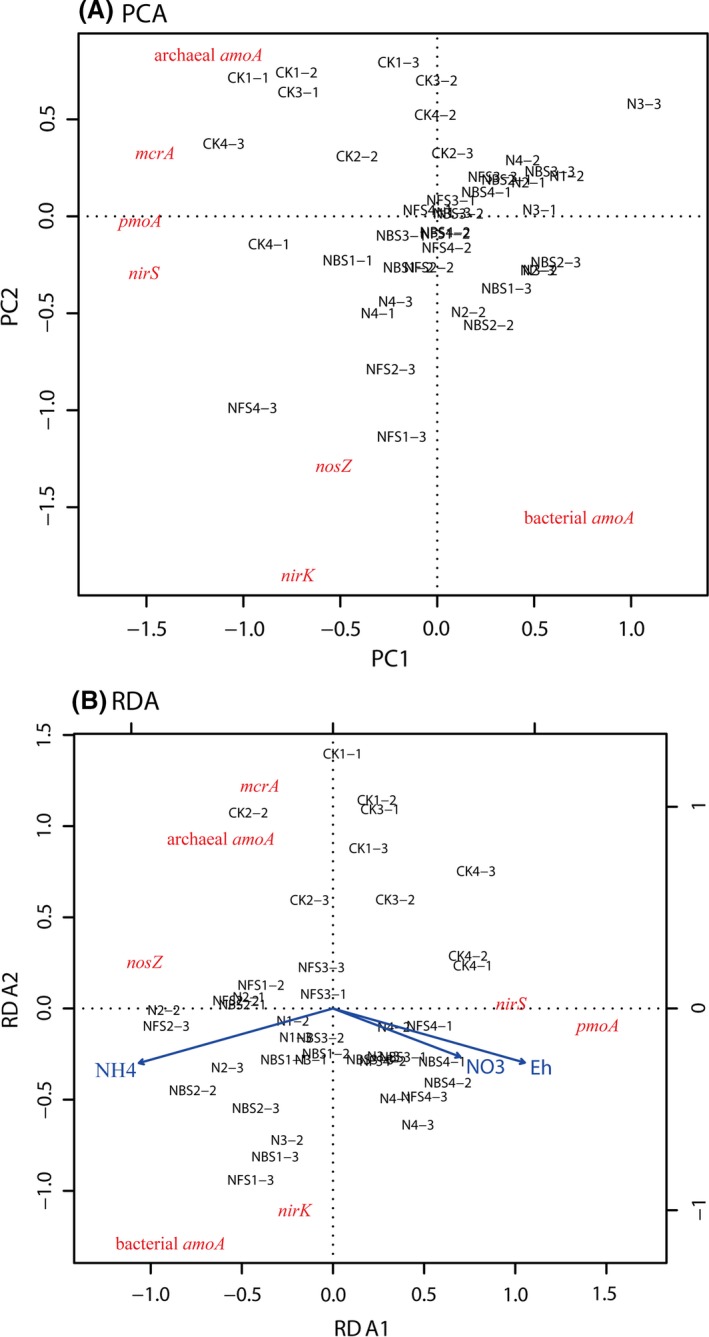
Outputs of multivariate analysis for the abundance of functional genes: (A) Principal component analysis (PCA), (B) Redundancy analysis (RDA). CK: without fertilizers, N: urea, NFS: urea + fresh straw, NBS: urea + burnt straw. The “1” in “CK1‐2” indicates this sample was collected on the first sampling day, and the “2” in “CK1‐2” indicates this is the second replicate.

Result of PCA showed the first axis explained 32.83% of microbial variance and the second axis 24.96%. Samples of Treatment CK tended to cluster together, while samples of the other three treatments tended to cluster together. And this indicated the functional microbial composition in Treatment CK was significantly different from the one in the other three treatments, while there was no significant difference of the functional microbial composition among Treatment N, Treatment NFS, and Treatment NBS. The positions of archaeal *amoA* and *mcrA* were closer to the cluster of Treatment CK, which indicated urea application decreased the gene abundance of archaeal *amoA* and *mcrA* (Fig. 3A). The positions of bacterial *amoA*,* nirK,* and *nosZ* were closer to the cluster of Treatment N, Treatment NFS, and Treatment NBS, which indicated urea application increased the gene abundances of bacterial *amoA, nirK,* and *nosZ*. Result of RDA showed that the three environmental factors explained 25.02% of microbial variance. And Monte Carlo permutation test indicated all of them were significant (P_Eh_ = 0.001; $P_{{\mathrm{NH}}_{4}^{+}}$ = 0.001; $P_{{\mathrm{NO}}_{3}^{-}}$ = 0.002). The clustering pattern of samples in the RDA plot was similar to that in the PCA plot (Fig. 3B). The vectors of environmental factors point to the cluster of Treatment N, Treatment NFS, and Treatment NBS, which indicates urea application increased the soil Eh, soil ${\text{NH}}_{4}^{+}$, and ${\text{NO}}_{3}^{-}$ contents. Besides, soil Eh was negatively related to soil ${\text{NH}}_{4}^{+}$ but positively related to soil ${\text{NO}}_{3}^{-}$. The vector of soil Eh point to the *nirS* and *pmoA*, which indicated higher soil Eh increased the abundances of *nirS* and *pmoA*. The positions of *nosZ* and archaeal *amoA* were on the reverse direction of the soil Eh vector, which indicated higher soil Eh decreased the abundances of *nosZ* and archaeal *amoA*.

## Discussion

### Effects of fertilization on related soil microbial community

In this study, urea application significantly changed the functional microbial composition, regardless of addition of straw or not (Fig. 3, Table 3). Nitrogen is often considered to be the element most often limiting to net primary production in terrestrial ecosystems (Vitousek and Howarth 1991). Plants and soil microorganisms are both limited by inorganic nitrogen, even on relatively fertile sites (Kaye and Hart 1997; Schimel and Weintraub 2003). The nitrogen limitation may be intensified by denitrification in rice soils. This may explain the significant effect of urea application on the functional microbial composition.

Urea application tended to increase the soil ${\text{NH}}_{4}^{+}$ and ${\text{NO}}_{3}^{-}$ contents (Fig. 1I–J). Methanogenesis can be directly lowered by stimulating competing nitrate‐reducing bacteria, which can also produce toxic compounds (${\text{NO}}_{2}^{-}$, NO, N_2_O) (Achtnich et al. 1995). Indirectly, stimulation of plant growth by ammonium fertilizers can stimulate methanogens by increasing very labile root exudates (Lu and Conrad 2005). In this study, application of urea significantly decreased the abundance of *mcrA* gene (Figs. 1A, 3, Table 2), which might be resulted from the inhibiting effect of high ${\text{NH}}_{4}^{+}$‐N concentration on methanogens (Steinhaus et al. 2007; Zhang et al. 2014). It was thought that methane oxidation by methanotrophs in the rhizosphere of rice was nitrogen‐limited, and ammonium‐based fertilization was the stimulator of methane consumption (Bodelier et al. 2000). However, at the enzyme level, it was assumed that ammonium‐based fertilization would inhibit methane oxidation owing to competitive inhibition of methane oxidation (Gulledge et al. 1997). In this study, application of urea did not significantly affect the abundance of *pmoA* gene, which might be explained as that the inhibiting and stimulating effects of urea application on the methaotrophs were almost equal. Straw addition had little effect on the gene abundance of *pmoA* and *mcrA* in this study (Figs. 1A and B, 3). This might be resulted from microbial functional redundancy of methanogen and methanotrophs (Konopka et al. 2015; Souza et al. 2015).

Urea application significantly increased the gene abundance of bacterial *amoA* but significantly decreased the gene abundance of archaeal *amoA* (Figs. 1C and D, 3, Table 2). However, it was also reported that application of urea both significantly increased the abundance of bacterial and archaeal *amoA* genes in the rice soils (Song and Lin 2014). Previous studies show ammonia‐oxidizing archaea (AOA) and ammonia‐oxidizing bacteria (AOB) response differently to soil pH and inorganic fertilizer (He et al. 2007; Wu et al. 2011). And they may have different niches in ammonia oxidation (Schleper 2010), which might result their contrary responses to urea application. The effects of application of urea on the abundance of *nirK* and *nirS* genes were also different (Figs. 1E and F, 3). Previous study reported that the *nirK*‐containing denitrifiers and *nirS*‐containing denitrifiers have different niches (Philippot et al. 2009; Chen et al. 2010). Hence, they respond to higher urea application differently as was observed in this study. However, straw incorporation had little effect on the functional genes of nitrogen transformation except *nosZ* (Figs. 1C–G, 3, Table 2). Previous study indicated microbes containing *nosZ* gene were highly sensitive to soil Eh variation (Richardson et al. 2009). And straw decomposition can intensify anaerobic conditions, which may favor the N_2_O reducers and result into the higher abundance of *nosZ* gene in Treatment NFS.

### Effects of fertilization on CH_4_ and N_2_O fluxes

It is thought CH_4_ emission can be largely affected by methanogenic (*mcrA*) and methanotrophic (*pmoA*) microbes (Conrad 2007). However, in this study, the correlation between CH_4_ emission and the abundance of *mcrA* and *pmoA* genes was not significant (Table 1). The gene abundance of *mcrA* and *pmoA* in Treatment NFS tended to be lower than the one in Treatment CK (Fig. 1A and B), while CH_4_ emission was significantly higher in Treatment NFS than the one in Treatment CK (Figs. 1K, 2C). Concentration and type of organic matter are one of the determining factors for CH_4_ production (Dalal et al. 2008). Straw incorporation may highly stimulate the activity of methanogen by providing fresh organic matters (Glissmann and Conrad 2000; Glissmann et al. 2001). Most of the carbon contained in the straw was lost during burning (Heard et al. 2006), leaving limited labile organic carbon in straw ash as substrate for methanogen. Therefore, CH_4_ emission was much higher in Treatment NFS than the one in Treatment NBS (Fig. 2A).

In this study, the correlation between N_2_O emission and the abundance of related functional genes was not significant (Table 1). However, N_2_O is produced in soils mainly through nitrification and denitrification (Braker and Conrad 2011). Application of urea can stimulate the activity of ammonia‐oxidizing microbes by providing abundant substrates for them (Wu et al. 2011), and denitrification may also be stimulated by more nitrate (Fig. 1I and J). And in this study, application of urea significantly increased the gene abundance of bacterial *amoA* (Figs. 1D, 3B, Table 2). This may explain the application of urea without straw significantly increased N_2_O emission (Fig. 2B), which was consistent with previous studies (Ma et al. 2007; Pittelkow et al. 2014). N_2_O emission also tended to be higher in the Treatment NFS and Treatment NBS than the one in Treatment CK (Fig. 2B). However, N_2_O emission in Treatment NFS and Treatment NBS tended to be lower than the one in Treatment N (Fig. 2B). The abundance of *nosZ* gene in Treatment NFS and Treatment NBS also tended to be higher than the one in Treatment N (Fig. 1G). A recent meta‐analysis study also reveals that straw incorporation decreases N_2_O emission by 15.2 ± 1.1% in paddy soils (Liu et al. 2014).

### Effects of time on related soil microbial community and CH_4_ & N_2_O fluxes

In this study, time did not significantly change the functional microbial composition, as samples collected on the 4 days are clustered together (Fig. 3). However, time significantly affects the abundances of *pmoA*, archaeal *amoA*,* nirS,* and *nosZ* genes (Table 2). This might be resulted from midseason aeration. Midseason aeration can significantly increase the soil Eh (Fig. 1H). Methanotrophic bacteria and AOB are all strictly aerobic microorganisms (Bodelier et al. 2000; Könneke et al. 2005), while N_2_O reducers were strictly anaerobic microorganisms (Richardson et al. 2009). And they are all very sensitive to soil Eh variation. Soil ${\text{NH}}_{4}^{+}$ and ${\text{NO}}_{3}^{-}$ contents can be affected by soil Eh (Fig. 3B). Soil ${\text{NO}}_{3}^{-}$ content was also significantly higher after midseason aeration (Fig. 1J), which may result in the higher abundance of *nirS* gene for providing more substrates (Fig. 1E). Higher abundance of *pmoA* gene may explain the lower CH_4_ emission after midseason aeration (Fig. 1B and K). Higher abundance of *nirS* gene and lower abundance of *nosZ* gene may explain the higher N_2_O emission after midseason aeration (Fig. 1E, G, L). However, the correlation between the abundance of functional genes and CH_4_ and N_2_O emissions was not significant (Table 1). Soil Eh is a key factor controlling CH_4_ and N_2_O emissions (Hou et al. 2000; Yu et al. 2001). The correlation between CH_4_ emission and the soil Eh was significant (Table 1). The negative correlation between CH_4_ and N_2_O emissions over time in a midseason aerated rice paddy field was also observed in previous studies (Cai et al. 1997; Ma et al. 2007).

## Conclusions

The data acquired through this study indicate that in rice paddy fields, application of urea significantly changed the functional microbial composition, regardless of addition of straw or not. Application of urea significantly decreased the gene abundances of archaeal *amoA* and *mcrA*, but it significantly increased the gene abundance of bacterial *amoA*. CH_4_ emission was significantly increased by fresh straw incorporation. Incorporation of burnt straw tended to increase CH_4_ emission, while the urea application had no obvious effect on CH_4_ emission. N_2_O emission was significantly increased by urea application, and fresh or burnt straw incorporation tended to decrease N_2_O emission. The functional microbial composition did not change significantly over time, although the abundances of *pmoA*, archaeal *amoA*,* nirS* and *nosZ* genes changed significantly. The change of CH_4_ emission showed an inverse trend with the one of the N_2_O emissions over time. To some extent, the abundance of some functional genes in this study can explain CH_4_ and N_2_O emissions. However, the correlation between CH_4_ and N_2_O emissions and the abundance of related functional genes was not significant. Environmental factors, such as soil Eh, may be more related to CH_4_ and N_2_O emissions.

## Conflict of Interest

None declared.

## Supporting information


**Table S1.** Schedule of fertilizer application and water management during the rice season.
**Table S2.** Primers used for real‐time PCR to measure the abundance of soil microorganisms.
**Table S3.** The limitations of the primers used in this study.
**Table S4.** The details of redundancy analysis in this study (Ren et al., 2014).
