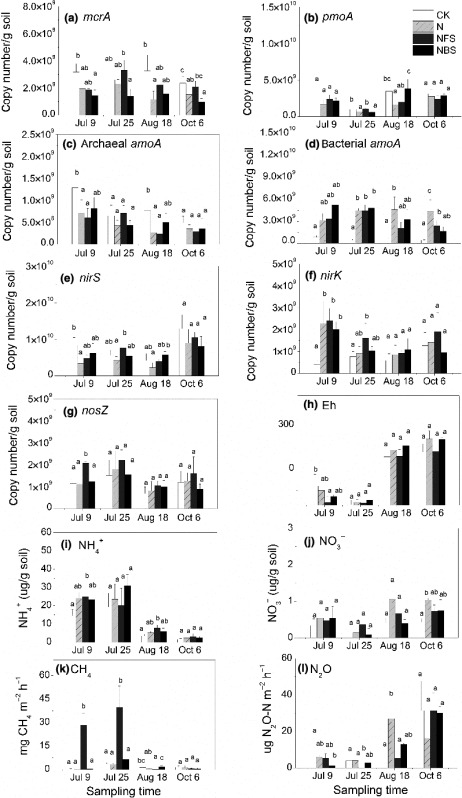# Corrigendum

**DOI:** 10.1002/ece3.3769

**Published:** 2018-01-08

Fan, X., Yu, H., Wu, Q., Ma, J., Xu, H., Yang, J., Zhuang, Y. (2016). Effects of fertilization on microbial abundance and emissions of greenhouse gases (CH_4_ and N_2_O) in rice paddy fields. *Ecology and Evolution*, 6(4), 1054–1063. https://doi.org/10.1002/ece3.1879

Page 1058, Figure 1a: The scale of this figure related to the abunance of *mcrA* gene should be revised from ×10^11^ to ×10^9^. Just as below.