# Supplementary material for: Effects of fertilization on microbial abundance and emissions of greenhouse gases (CH 4 and N2O) in rice paddy fields
Source: Ecol Evol. 2016 Jan 22;6(4):1054–63. doi: 10.1002/ece3.1879 (PMC4722792; doi:10.1002/ece3.1879)
Supplement: Supplementary file 1 — Table S1. Schedule of fertilizer application and water management during the rice season. Table S2. Primers used for real‐time PCR to measure the abundance of soil microorganisms. Table S3. The limitations of the primers used in this study. Table S4. The details of redundancy analysis in this study (Ren et al., 2014). [file ECE3-6-1054-s001.docx]

Supporting information

**Table S1.** Schedule of fertilizer application and water management during the rice season.

| Fertilizer application | Date | Water management | Date |
| --- | --- | --- | --- |
| Organic fertilizer: wheat straw, 4.8 t ha^-1^ | 26 Jun | Continuous flooding | 26 Jun |
| Basal N-fertilizer: Urea, 150 kg N ha^-1^ | 26 Jun | Midseason aeration | 30 Jul |
| Tillering N-fertilizer: Urea, 75kg N ha^-1^ | 23 Jul | Alternation of drying and wetting | 28 Aug |
| Panicle N-fertilizer: Urea, 75 kg N ha^-1^ | 17 Aug | Final drainage | 13 Oct |

**Table S2.** Primers used for real-time PCR to measure the abundance of soil microorganisms.

| Microorganisms | Target gene | Primer sets | References |
| --- | --- | --- | --- |
| Methanogens | *mcrA* | 5′-GCMATGCARATHGGWATGTC-3′ | Hales et al.1996 |
|  |  | 5′-TGTGTGAASCCKACDCCACC-3′ |  |
| Methanotrophs | *pmoA* | 5′-GGNGACTGGGACTTCTGG-3′ | Costello et al.1999 |
|  |  | 5′-CCGGMGCAACGTCYTTACC-3′ |  |
| Nitrifying archaea, bacteria | archaeal *amoA* | 5′- STAATGGTCTGGCTTAGACG-3′ | [Francis et al. 2005](#_ENREF_3) |
|  |  | 5′- GCGGCCATCCATCTGTATGT-3′ |  |
|  | bacterial *amoA* | 5′-GGGGTTTCTACTGGTGGT-3′ | [Rotthauwe et al. 1997](#_ENREF_14) |
|  |  | 5′-CCCCTCKGSAAAGCCTTCTTC-3′ |  |
| Denitrifying bacteria | *nirK* | 5′-ATCATGGTSCTGCCGCG-3′ | Hallin and Lindgren 1999 |
|  |  | 5′-GCCTCGATCAGRTTGTGGTT-3′ |  |
|  | *nirS* | 5′-GASTTCGGRTGSGTCTTGA-3′ | Michotey et al. 2000 |
|  |  | 5′-GTSAACGTSAAGGARACSGG-3′ | Throbäck et al. 2004 |
|  | *nosZ* | 5′-CGYTGTTCMTCGACAGCCAG-3′ | Kloos et al. 2001 |
|  |  | 5′-CGSACCTTSTTGCCSTYGCG-3′ | Throbäck et al. 2004 |

**Table S3.** The limitations of the primers used in this study.

| Target gene | Limitations of the primers | References |
| --- | --- | --- |
| *mcrA* | The primer set may fail to detect *Methanosarcinaceae*, an unidentified rice field soil *mcrA* cluster and *Methanobacteriaceae* mrtA | Juottonen et al. 2006 |
| *pmoA* | The primer set may fail to detect the bacteria and archaea of anaerobic oxidation of methane, such as *Candidatus Methylomirabilis oxyfera*, anaerobic methanotrophic archaea | Cui et al. 2015,  Bourne et al. 2001 |
| archaeal *amoA* | The primer set is with amplification bias of *Nitrososphaera* 1.1 and *Nitrososphaera* 54d9 | Meinhardt et al. 2015 |
| bacterial *amoA* | The primer set is with amplification bias of *Nitrosospira* 2 | Meinhardt et al. 2015 |
| *nirK* | The primer set may fail to detect the bacteria and archaea with copper-containing nitrite reductase in the Clusters II–IV | Wei et al. 2015 |
| *nirS* | The primer set may fail to detect the bacteria with cytochrome cd1-containing nitrite reductase in Clusters II–III | Wei et al. 2015 |
| *nosZ* | The primer set may fail to detect *nosZ* in non-Proteobacteria strains, including Firmicutes (Gram-positive) and Bacteroidetes | Jung et al. 2013 |

**Table S4.** The details of redundancy analysis in this study (Ren et al., 2014).

|  | Details |
| --- | --- |
| Samples | a. Each sample characterized as a combination of the different genes analyzed, and the combination was described as functional microbial composition;  b. Abundances of genes was expressed in cope number/g soil and normalized before analysis;  c. The distance between samples indicates the dissimilarity of functional microbial composition between samples, and cluster indicates the similarity of functional microbial composition between samples. |
| Functional genes | The shorter distance between functional gene and the samples indicates the samples contain higher abundance of it. |
| Environmental vectors | a. The environmental vectors point in the general direction of maximum environmental change across the diagram;  b. Their lengths are approximately proportional to the rate of change in that direction;  c. The angles between vectors indicate correlations between individual environmental variables. A positive correlation is reflected by an angle smaller than 90° and the smaller the angle, the closer the positive relation of the two variables. A negative correlation is suggested by an angle larger than 90°, while little relation between two variables is indicated by 90°. |

References

Bourne DG, McDonald IR, Murrell JC (2001) Comparison of pmoA PCR primer sets as tools for investigating methanotroph diversity in three danish soils. *Applied and Environmental Microbiology*, **67,** 3802–3809.

Costello AM, Lidstrom ME (1999) Molecular characterization of functional and phylogenetic genes from natural populations of methanotrophs in lake sediments. *Applied and Environmental Microbiology*, **65**, 5066–5074.

Cui M, Ma A, Qi H et al. (2015) Anaerobic oxidation of methane: an “active” microbial process. *MicrobiologyOpen*, **4(1)**, 1 – 11.

Francis CA, Roberts KJ, Beman JM et al. (2005) Ubiquity and diversity of ammonia-oxidizing archaea in water columns and sediments of the ocean. *Proceedings of The National Academy of Sciences of The United States of America*, **102**, 14683-14688.

Hales BA, Edwards C, Ritchie DA et al. (1996) Isolation and identification of methanogen-specific DNA from blanket bog peat by PCR amplification and sequence analysis. *Applied and Environmental Microbiology*, **62**, 668–675.

Hallin S, Lindgren PE (1999) PCR detection of genes encoding nitrite reductase in denitrifing bacteria. *Applied and Environmental Microbiology*, **65**, 1652–1657.

Jung J, Choi S, Jung H et al. (2013) Primers for amplification of nitrous oxide reductase genes associated with Firmicutes and Bacteroidetes in organic-compound-rich soils. *Microbiology*, **159**, 307–315.

Juottonen H, Galand PE, Yrjälä K. (2006) Detection of methanogenic Archaea in peat: comparison of PCR primers targeting the mcrA gene. *Res. Microbiol,* **157**, 914–921.

Kloos K, Mergel A, Rosch C et al. (2001) Denitrification within the genus Azospirillum and other associative bacteria. *Australian Journal of Plant Physiology*, **28**, 991–998.

Meinhardt KA, Bertagnolli A, Pannu MW et al. (2015) Evaluation of revised polymerase chain reaction primers for more inclusive quantification of ammonia-oxidizing archaea and bacteria. *Environmental Microbiology Reports*, **7(2)**, 354–363.

Michotey V, Mejean V, Bonin P (2000) Comparison of methods for quantification of cytochrome *cd*_1_-denitrifying bacteria in marine samples. *Applied and Environmental Microbiology*, **66**, 1564–1571.

Ren J, Gersonde R, Esper O et al. (2014) Diatom distributions in northern North Pacific surface sediments and their relationship to modern environmental variables. *Palaeogeogr. Palaeoclimatol. Palaeoecol.*, **402,** 81–103.

Rotthauwe J, Witzel K, Liesack W (1997) The ammonia monooxygenase structural gene amoA as a functional marker: molecular fine-scale analysis of natural ammonia-oxidizing populations. *Applied and Environmental Microbiology*, **63**, 4704-4712.

Throbäck IN, Enwall K, Jarvis A et al. (2004) Reassessing PCR primers targeting nirS, nirK and nosZ genes for community surveys of denitrifying bacteria with DGGE. *FEMS Microbiology Ecology*, **49**, 401–417.

Wei W, Isobe K, Nishizawa T et al. (2015) Higher diversity and abundance of denitrifying microorganisms in environments than considered previously. *The ISME Journal*, **9**, 1954–1965.
